# Effect of Bed Characters on the Direct Synthesis of Dimethyldichlorosilane in Fluidized Bed Reactor

**DOI:** 10.1038/srep08827

**Published:** 2015-03-06

**Authors:** Pan Zhang, Ji H. Duan, Guang H. Chen, Wei W. Wang

**Affiliations:** 1 College of Electromechanical Engineering, Qingdao University of Science and Technology, Qingdao 266061, China; 2 College of Chemical Engineering, Qingdao University of Science and Technology, Qingdao 266042, China

## Abstract

This paper presents the numerical investigation of the effects of the general bed characteristics such as superficial gas velocities, bed temperature, bed heights and particle size, on the direct synthesis in a 3D fluidized bed reactor. A 3D model for the gas flow, heat transfer, and mass transfer was coupled to the direct synthesis reaction mechanism verified in the literature. The model was verified by comparing the simulated reaction rate and dimethyldichlorosilane (M2) selectivity with the experimental data in the open literature and real production data. Computed results indicate that superficial gas velocities, bed temperature, bed heights, and particle size have vital effect on the reaction rates and/or M2 selectivity.

Dimethyldichlorosilane(M2) is one of most important organic silicon monomers, which is synthesized commercially by the direct process in which gaseous methyl chloride(MeCl) is reacted with metallurgical grade silicon powder in the presence of catalytic amounts of copper with various promoters^1^. The principle reaction can be represented as follows^2^.
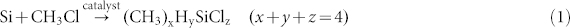
One of the most features of the direct synthesis process is the great number of by-products resulting from this reaction. The reaction system has high complexity. The reaction rate and the selectivity depend on a large number of factors some of which have not yet been identified. Because of the superior properties and good application future of the organosilicon materials, the subject has received a great deal of attention.

Numerous reports have appeared on the kinetics of the reaction^3^,^4^. The influence of the contents of the catalysts^5^ and promoters such as zinc^6^, tin^1^,^7^, phosphorus, stannum^5^ and aluminum^2^, the structure of the catalysts^8^ on the reaction kinetics have been experimentally investigated in a flow reactor, a fixed bed reactor or a fluidized bed reactor. De Cooker et al.^9^,^10^ has studied the effect of the addition of hydrogen and oxygen on the product composition and an overall selectivity of the reaction. Acker and Bohmhammel^11^ conducted a thermodynamic study on the reaction system of the direct synthesis.

However, the reaction rate and the product selectivity are determined not only by the chemical reaction, but also by physical phenomena such as adsorption of MeCl, desorption of the reaction products, especial various transport processes^12^, which play a dominant role for the profile of the pressure, the reactant and reaction product concentration. Many fluidized bed characters such as superficial gas velocities, bed temperature, bed heights and particle size, have certainly the vital effect on the various transport processes, and then on the direct synthesis process. Ward et al^1^ also pointed out that the reactor type has an important effect on the direct synthesis process. With good transport character, it is usually thought that the reaction activity and M2 selectivity in the fluidized bed reactor (FBR) can be higher than that in the fixed-bed reactor (FXBR). But relevant more studies were only carried out under certain operating parameters^7^,^13^,^14^,^15^. The characters which influence the transport process, such as the particle size, superficial gas velocity and bed height, were less discussed.

It has been recognized that numerical models based on computational fluid dynamics (CFD), accounting for the interaction between gas-solid flows, heat and mass transfer and chemical reactions, can be of great help in the optimization of various complex processes. Besides, CFD numerical models may provide fundamental insights into the underlying physico-chemical processes^16^,^17^,^18^,^19^,^20^. The Eulerian-Eulerian model with kinetic theory of granular flow is the most applicable approach to compute gas-solid flow. Many authors have utilized laminar or turbulence model with kinetic theory of granular flow for modeling the hydrodynamics of gas-solid multiphase flow^21^,^22^. Both the laminar and turbulence models can be used to obtain the main features of the flow in the fluidized bed and/or riser^22^,^23^,^24^. To account for the interactions of turbulence between the gas and solid phase, Dasgupta, Jackson, and Sundaresan^25^, Bolio and Sinclair^26^, Hrenya and Sinclair^27^ and Simonin^28^ have reported the use of turbulence models for gas-solids flows. Zheng et al.^29^ proposed the k-ε-k_p_-ε_p_-Θ model to simulate the turbulent gas–particle flow in a riser reactor and the predictions showed satisfactory agreements with experimental data. The k-ε turbulence model was even applied to account for the turbulence induced by the dispersed phases in three-phase fluidized bed^30^. The turbulence model developed by Simonin's group^28^,^31^ was implemented in CFD code MFIX (Multiphase Flow with Interphase eXchanges; www.mfix.org)^32^ developed at the National Energy Technology Laboratory. This model was used to predict reasonably well dilute gas-solids flows with appropriate boundary conditions^33^.

The main object of the present paper is to investigate the influence of the general bed characteristics on the direct synthesis process in the fluidized bed reactor. CFD code MFIX combined with a validated chemistry model was used to describe the direct synthesis process in which M2 is prepared.

## Model

### Reactor Geometry

**Figure 1:**
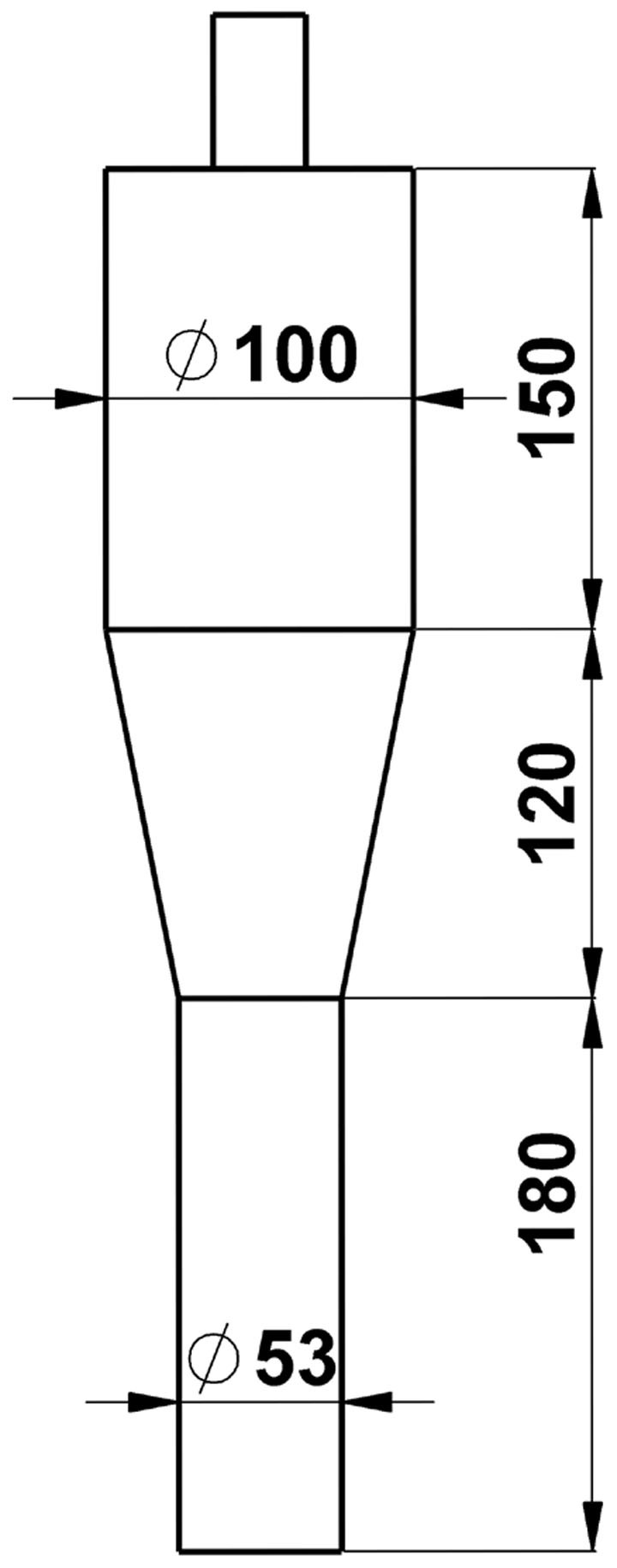
Schematic diagram of the reactor(unit: mm).

A three-dimensional (3D) model coupled with synthesis reaction is considered for the fluidized bed reactor illustrated in Fig. 1. The dimension of the reaction zone is 53 mm (diameter) × 180 mm (height). The expanding section has 100 mm diameter and 150 mm height. There is a 120-mm-height cone section between them.

MeCl gas enters the reactors at the bottom. The gaseous MeCl is reacted with metallurgical grade silicon powder. Finally, the reactant and product gases leave the reactors through the outlet, which is fixed at 3.0 atm. Catalyst was not considered in the flow model, and its effect on the reaction was embodied by the value of the reaction activation energy.

### Governing Equations

A multiphase hydrodynamic code, MFIX^32^,^34^, based on the Euler-Euler model which treats the fluid and the solids as two interpenetrating continua, was used to describe the gas-solid flow model. The model can give detailed information on the temporal and spatial evolutions of local void fractions, gas and particle velocities, species gas fractions and chemical reaction rates.

For our model, the gas is considered as the primary phase, whereas the solid phase is considered as secondary phase. The primary and secondary phases are linked by tracking the phase volume fractions in a finite-volume frame. These volume fractions are assumed to be functions of space and time. By definition, the volume fractions of all of the phases must sum to unity:
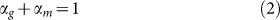
where *α* is the volume fraction, the subscript *g* and *m* indicate gas and solid phase, respectively. The multiphase CFD model for gas–solid flow is described briefly below. More details can be found elsewhere^32^,^34^.

Continuity for phase *l* (*l* = *g* or *m*):
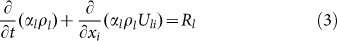
where *ρ* stands for density, *U* stands for velocity component, *x* stands for coordinate, the subscript *i* stands the *ith*-direction and *R_l_* are the inter phase mass transfer terms between the gas and solid phase due to surface chemistry at the gas–solid interface.

Momentum conservation for the gas phase:
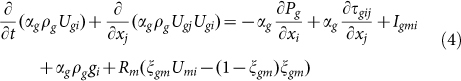


Here, *P_g_* is the gas phase pressure and *τ_gij_* is the second-order stress tensor for the gas phase, which is expressed as:
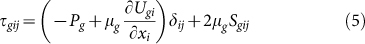
where the gas phase deformation rate 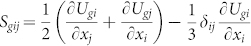, *μ* is the bulk viscosity and *δ_ij_* is the Kronecker Delta. *I_gm_* is an interaction force representing the momentum transfer between the gas and the solid phase, and *g* is the gravity. The last term of the left hand side stands for the momentum transfer between the gas and the solid phase duo to mass transfer from solid phase to the gas phase. *ξ_gm_* is defined as
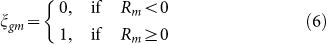


Momentum conservation for the solid phase:
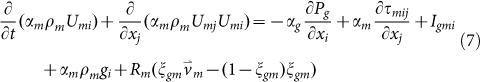
where the solid phase tress tensor *τ_mij_* is expressed as:
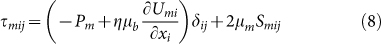
where the solid phase deformation rate 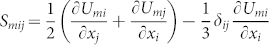, the solid pressure, *P_m_* = *α_m_ρ_m_*Θ*_m_*(1 + 2(1 + *e_mm_*)*α_m_g*_0,*mm*_), Θ*_m_* is the granular temperature (solids fluctuating energy), which is calculated by an algebraic granular temperature equation. The bulk viscosity, 
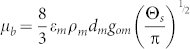
, 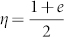. *e* is the restitution coefficient. The shear viscosity *μ_m_* consists of tow parts: the collisional part *μ_m,col_* and the kinetic part *μ_m,kin_*.
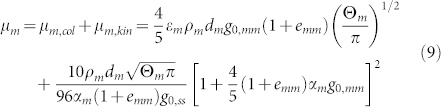


Turbulence modeling in the continuous phase:
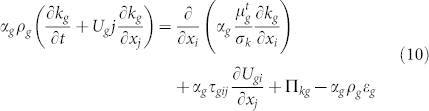

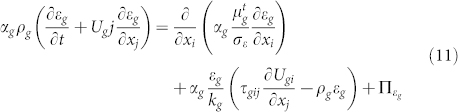
where 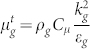, Π*_kg_* = *β*(*k_gm_* − 2*k_g_*), 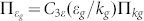. *σ_k_*, *σ_ε_*, *C*_1*ε*_ and *C*_2*ε*_ are constant parameters^35^. It is a standard k-epsilon model modified to account for the presence of a particle phase. Such an approach has been successfully used in many turbulent two-phase flows^33^,^36^,^37^,^38^. Benyahia et al.^39^ proposed that the use of proper gas phase turbulence interaction with the particulate phase has important effect on the flow in the dense particle flows.

Turbulence modeling of the solid phase:
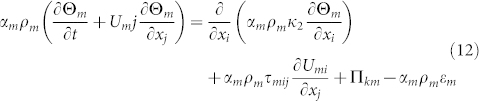

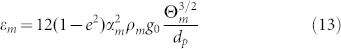
where, Π*_km_* = *β*(*k_gm_* − 3Θ*_m_*), *η_t_* is the Ratio between the Lagrangian integral time scale and the particle relaxation time. The solids granular conductivity is expressed,
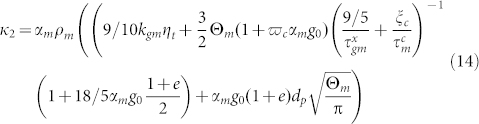
where 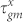 is particle relaxation time, 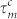 is collisional time-scale, 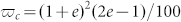, *ξ_c_* = (1 + *e*)(49 − 33*e*)/100^35^.

Energy balance equation for gas phase:
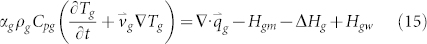
where *C_pg_* is the specific heat capacity of the gas phase, *T* is the temperature, 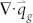 is the gas-phase conductive heat flux, *H_gm_* describes fluid–solids interphase heat transfer between the gas and solid phase, and Δ*H_g_* is the heat of reaction in the gas phase. The last term accounts for the heat loss to the wall.

Energy balance equation for gas phase:
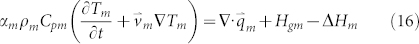
where *C_pm_* is the specific heat capacity of the solid phase, 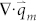 is the solid-phase conductive heat flux, *H_gm_* describes fluid–solids interphase heat transfer between the gas and solid phase, and Δ*H_m_* is the heat of reaction in the solid phase.

The gas and solid phases may contain an arbitrary number of chemical species n. The species conservation equations considering the accumulation, convection, and rate of reaction for the gas or solid phase is
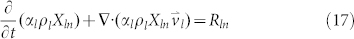
where *X_ln_* is the mass fraction and *R_ln_* is the rate of formation of the phase *l*(*l* = *g* or *m*), species n. The diffusion flux is neglected compared to the convection flux.

### Constitutive Relations

Constitutive relations are needed to close governing relations. A simple Newtonian closure is used for the gas-phase stress tensor. The gas-solid momentum exchange coefficient is adopted by Syamlal et al.^34^.

Two entirely different approaches are used to describe the stresses in two distinct flow regimes: a viscous shearing and a plastic regime^34^,^40^. Kinetic theory is used to calculate the solid-phase stress tensor in the viscous regime^41^. A granular temperature is described as the specific kinetic energy of the random fluctuating component of the particle velocity. The transport of the mixture granular energy can be solved and then the granular temperature for the solid phase can be obtained. Here, an algebraic expression for granular temperature Θ*_m_* is calculated^34^. The solid pressure, which describes the change in the total momentum transport of the motion of particles and their interactions^41^, consists of a collision and a kinetic term. The particle phase shear viscosity concludes three parts: collision viscosity, kinetic viscosity and frictional viscosity. The solids pressure and viscosity are computed as a function of granular temperature^34^,^41^,^42^,^43^,^44^. A solids stress tensor based on the critical state theory in the plastic flow regime was used^22^.

The conductive heat flux within the fluid phase is described by Fourier's law. The conductive heat flux in the solids phase is assumed to have a form similar to that in the fluid phase^45^. The heat transfer between the fluid and solids is assumed to be a function of the temperature difference. The heat-transfer coefficient is related to the particle Nusselt number proposed by Gunn^46^.

### Chemical Model

The two possible major chemical reactions occurred in the direct synthesis system are expressed in Eq.(18)–(19).
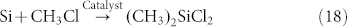

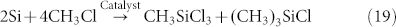


In this paper, a global scheme based on the adsorption and diffusion mechanism has been implemented to describe the chemistry of the direct synthesis. The diffusion of silicon atoms, through the solid phase onto active sites of the catalyst, where they can react with the MeCl, is considered as the rate determining step in the direct synthesis process^47^. Based on the above theory, the rate expression for the direct reaction has been assumed to be of the form^48^:
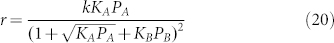
where *k* is the apparent rate constant, *K* are adsorption equilibrium constants for MeCl (A), and silane (B), (atm^−1^), *P* are partial pressure for MeCl (A), and silane (B), (atm)

The Arrhenius equation has been successfully used in chemical reaction kinetics to describe the temperature dependence of the reaction rate constant. Following the equation of Arrhenius, the apparent rate constant is the product of a pre-exponential factor and an exponential term (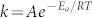), which was determined by fitting the Arrhenius equation using the experimental data by Voorhoeve et al.^14^. The activation energies of the Eq.(2)–(3) are 88.6 and 265.4 kJ/mol^49^,^50^, respectively. The change of the adsorption equilibrium constants with the temperature is expressed as *K_A_* = 94.1 − 10.89*T* + 9.74*E* − 4*T*^2,^^50^. *K_B_* changes slightly with temperature range of 280–340°C. Hence, it can be assumed that it is constant, and equal to about 0.500 atm^−1^.

### Numerical Method and Boundary Condition

The non-linear partial differential equations of the model are spatially discretized using a finite-volume technique on a staggered grid. A called Cartesian grid cut-cell technique has been implemented, which allows the definition of curved or sloping boundaries, instead of the usual stair-step representation. The utilization of the Cartesian cut-cell technique was described in the literature^51^. The grid size of 1.0 mm × 1.0 mm × 2.0 mm seemed sufficient to obtain expansion, height of fluctuation of the bed surface, frequency of fluctuation and MCl conversion that are independent of the grid resolution.

Compared to the solution of the hydrodynamics problem, the solution of the accompanying chemical reactions is not a trivial task because it gives the important chemical source terms in the aforementioned governing equations. Especially for the surface reaction in the study, the transport process (diffusion) that carries species to and from the surface may be comparable in rate to that of reaction at the surface. Transport and reaction have to be solved simultaneously as a set of nonlinear algebraic equations at each node on the reacting surface. The numerical stiffness of the multi-dimensional multi-species transport coupled synthesis chemistry models leads to poor convergence, excessive computation time, and unreliable predictions. The stiff integration of the system of ODEs is performed using the ODE solver ODEPACK (LSODA Fortran double-precision subroutine)^52^. An appropriate time step value for transport and for reaction was chosen for the sake of accuracy and stability of the MFIX code.

The segregated solution algorithm has been selected. For the pressure–velocity coupling, the SIMPLE (Semi-Implicit Method for Pressure-Linked Equations) method was used. Convective fluxes are approximated with the second-order accurate Superbee spatial scheme bounded TVD-scheme avoiding the excessive numerical diffusion. For pressure, linear discretization was used. A convergence criterion of 1 × 10^−06^ was used for continuity, momentum, energy and species transport equations.

At the column entrance, the velocity distribution is considered uniform. The velocity value was adjusted to correspond to the particle size. The gas feed is assumed to be uniform at 510 K. The reactor wall held at a fixed temperature condition. A no slip for gas and a free slip for solid are imposed on the side walls. The pressure outlet condition is applied at the outlet of the reactor.

## Results and Discussion

### Model Validation

The comparisons between the reaction rates and the M2 selectivity modeled and that under similar conditions in the literature are presented in Table 1. In our reaction model, the obtained products only consist of methyltrichlorosilane (CH_3_SiCl_3_, M1), M2, and trimethylchlorosilane ((CH_3_)_3_SiCl, M3). The M2 selectivity is defined as:
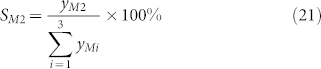
where *y_Mi_* is the mole fraction of *Mi* (*i* = 1,2,3) at the outlet of the reactor.

The relative errors between the modeled reaction rate and the ones in the FBR are less than 26.4%. The modeled reaction rate and M2 selectivity are more 7.9% and less 2.3% than the full-scale plant data, respectively. The maximum relative error between the modeled M2 selectivity and the data in the literature is 11%. It should be concluded that the proposed model is useful for describing the synthesis process of the methylchlorosilae in fluidized bed reactor.

### Effect of Reaction Temperature

**Figure 2:**
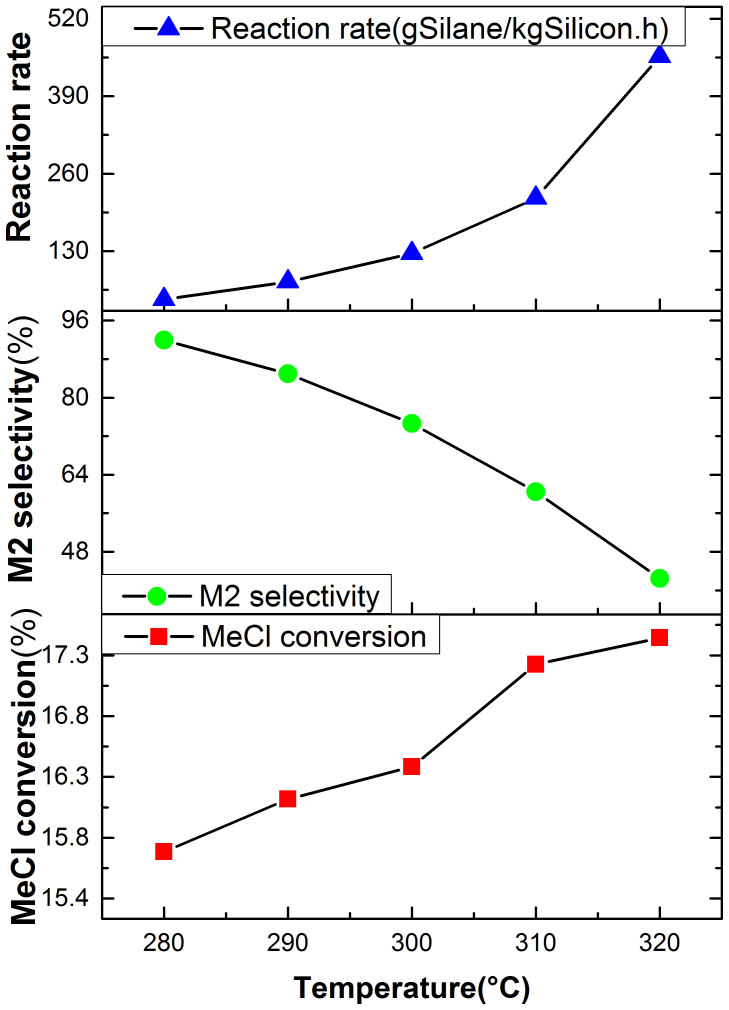
Effect of the temperature on reaction rate, M2 selectivity and MeCl conversion (Under pressure = 3.0atm, superficial gas velocity = 6.2 U_mf_, bed height = 12 cm and dp = 100 μm).

One of the most important parameters in the direct synthesis is the reaction temperature, which must be maintained at the required level on the surface of the contact mixture. Figure 2 shows the effects of temperature between 280 and 320°C on the reaction rate, the M2 selectivity and the MeCl conversion, which defined as 1 − *X_out_*/*X_in_* (*X_out_* = mass fraction of MeCl leaved (unreacted) the reactor. *X_in_* = mass fraction of MeCl was fed to the reactor). It can be seen that the M2 selectivity sharply decreases with the increase of the reaction temperature. The M2 selectivity is 91% at 280°C of the reaction temperature. And to 320°C, it is less than 50%. The reaction rate highly increases with the reaction temperature, especially after it exceeds 300°C. The change trend is similar to that proposed by Bablin et al.^7^, not to that by Ward et al.^1^, who thought that the temperature had no effect on product distribution. According to the Arrhenius equation, as the activation energy of Eq.(3) is much larger than that of Eq.(2), the rate of the reaction rate of Eq.(3) increases with the temperature would be much faster than that of Eq.(2). It should be the cause of that the M2 selectivity decreases with the increase of the reaction temperature. The MeCl conversion slightly increases with the increase of the temperature in the range of 15.7% to 17.4%.

The decomposition of the methyl groups generally becomes more pronounced with rising temperature, and the overall chlorine content of the products increases. This is economically unfavorable since M2 is much desirable than other chlorosilanes^3^. Then the optimum temperature will take into account the reaction rate and M2 selectivity.

### Effect of Particle Size

**Figure 3:**
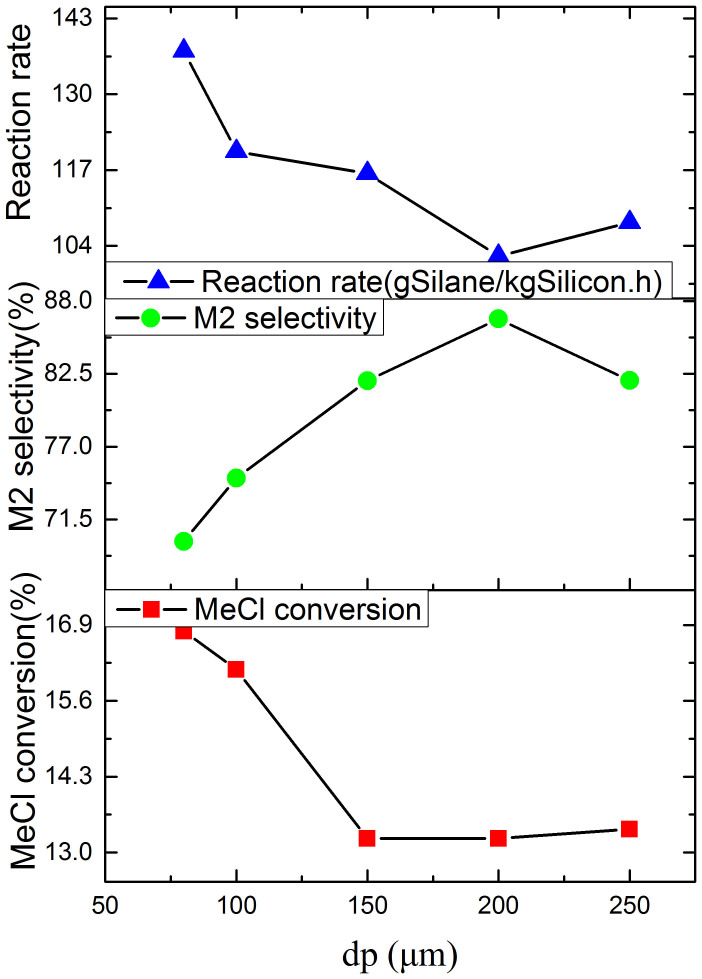
Effect of the particle diameter on reaction rate, M2 selectivity and MeCl conversion (Under pressure = 3.0atm, superficial gas velocity = 6.2 U_mf_, bed height = 12 cm and Temperature = 300°C).

**Figure 4:**
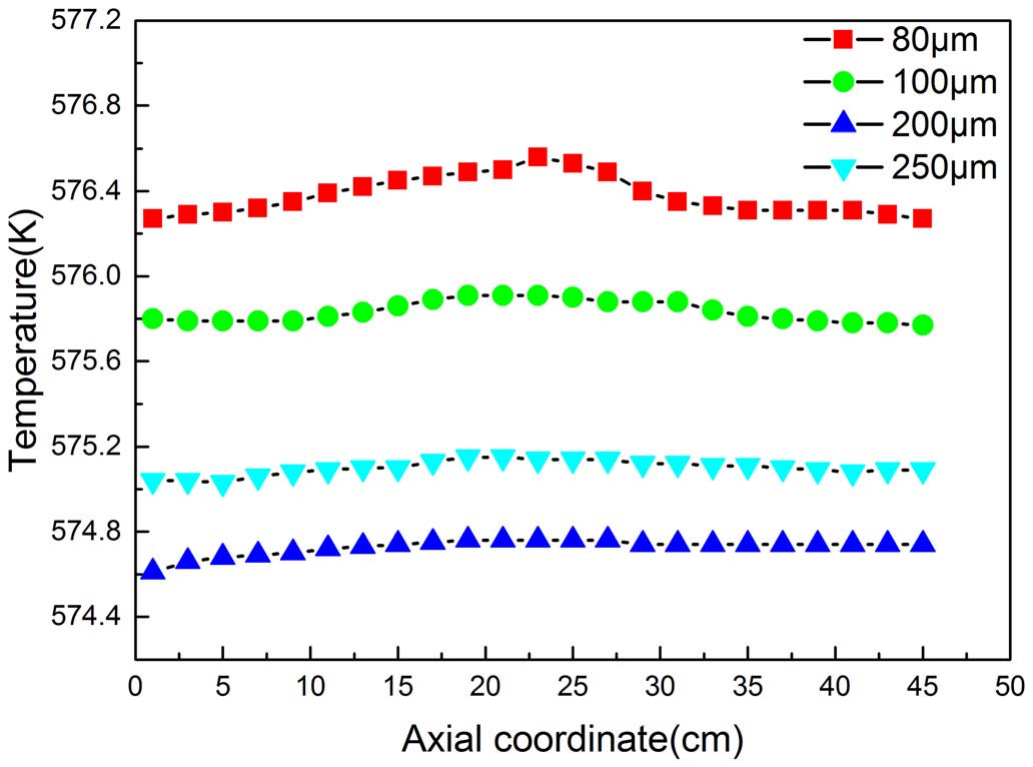
Effect of the particle diameter on the axial temperature distribution (Under pressure = 3.0atm, superficial gas velocity = 6.2 U_mf_, bed height = 12 cm and Temperature = 300°C).

Figure 3 shows the effects of the silicon particle diameter (dp) range from 80 to 250 μm on the reaction rate, the M2 selectivity and the MeCl conversion. It is can be seen that the M2 selectivity, firstly, increases with the increase of the particle diameter, and reaches the maximum value of 87% at about 200 μm diameter, and then decreases. In order to explain the phenomena, the axial average temperature distribution is plotted in the Fig. 4. It can be seen that there is a great difference of the average temperature for different diameter particles, which is highest for 80 μm particles, and is lowest for 200 μm particles. And there is a hump zone about at height range between 12 and 32 cm for all particle size in the reactor. The temperature peak value for 80 μm particles is the maximum, which reaches 576.5 K. The difference of the temperature peak values reaches 2.1°C between all diameter particles. It may be explained that the reactions are greatly exothermic, and smaller particles tend to aggregate, which worsened the heat transfer, and make the solid and gas temperature higher. According to the conclusion of the above section, the higher reaction temperature, the M2 selectivity is the lower.

Figure 3 also shows that the silicon particle diameter has an important effect on the reaction rate, especially when the particle diameter is less than 150 μm. The reaction rate is almost 1.4 times for 80 μm than that for 200 μm. Since the specific area (the surface area per unit volume) increases with the decrease of the particle diameter, the finer particles ensure a faster indication of the reaction between the methyl chloride and the silicon copper mixture. The above reaction rates are in accordance with this classical interface theory. The reaction rate almost reaches the smallest value of 103.5 at the 200 μm particle. The MeCl conversion, firstly, decreases with the increase of the particle diameter, and then almost tends towards the stable after the diameter of the particles exceeds 150 μm. It follows the same change trend as the reaction rate.

### Effect of Superficial Gas Velocity

**Figure 5:**
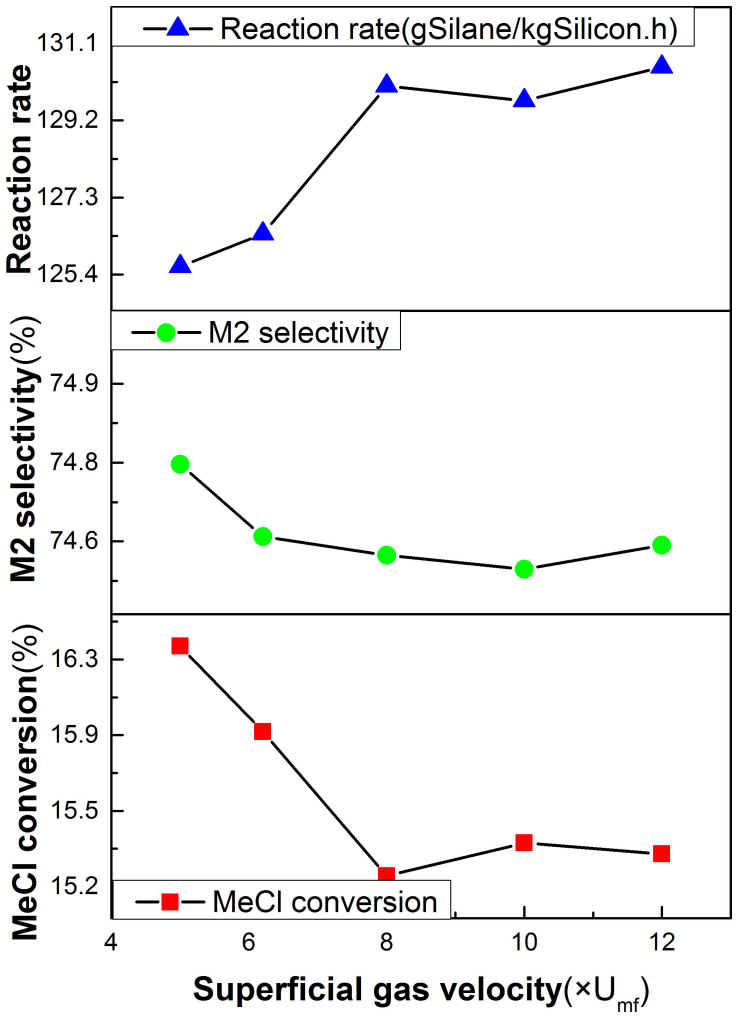
Effect of the superficial gas velocity on reaction rate, M2 selectivity and MeCl conversion (Under pressure = 3.0atm, dp = 100 μm, bed height = 12 cm and Temperature = 300°C).

Figure 5 shows the effects of the superficial gas velocity range from 5.2 to 12.0 times the minimum fluidization velocity (U_mf_) on the reaction rate, the M2 selectivity and the MeCl conversion. It is can be seen that the superficial gas velocity has less effect on the M2 selectivity, which almost keeps a constant value of 74.6%. However, the reaction rate almost lineally increases with the increase of the superficial gas velocity before it is less than 8 times U_mf_. The conclusion is consistent with the viewpoint of Bablin et al^7^, who proposed that the reaction is considered to be a pseudo first order in MeCl. The rate-limiting step of a fluidized bed reactor is generally determined either by the surface reaction kinetics or by mass transport^53^. The phenomenon should be explained that the process is limited by mass transport when the MeCl gas velocity is less than 8 times U_mf_. While the MeCl gas velocity exceeds 8 times U_mf_, the process is limited by the surface reaction kinetics. The higher concentration of the MeCl gas cannot further increase the reaction rate. When the MeCl gas velocity is less than 8 times U_mf_, the MeCl conversion and the reaction rate shows the opposite trend. This once again confirmed the above interpretation.

### Effect of Bed Height

**Figure 6:**
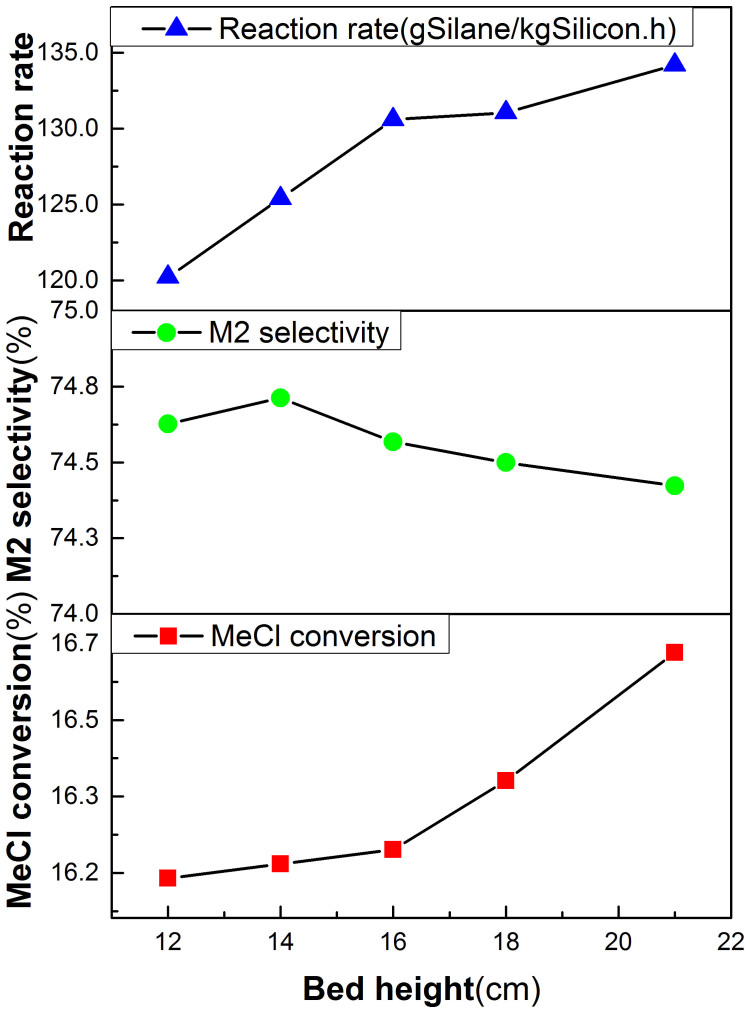
Effect of the bed height on reaction rate, M2 selectivity and MeCl conversion (Under pressure = 3.0atm, dp = 100 μm, superficial gas velocity = 6.2U_mf_, and Temperature = 300°C).

Figure 6 shows the effects of the bed height on the reaction rate, the M2 selectivity and the MeCl conversion. It is can be seen that the reaction rate, and the MeCl conversion have slightly increased by the average rates of 1.55 g/kg·h and 0.05% per cm bed height, respectively. It may be explained that the higher bed height, the more particle-surface area is provided for the gas-solid reaction. The M2 selectivity has slightly declined with the increase of the bed height.

Many researchers have studied or paid attention to the silicon conversion has a vital effect on the rate and the selectivity of the reaction^10^,^49^. Since further increases in silicon conversion would be attained on further extending the reaction time, we do not consider the influence of the silicon conversion. In fact, to some degree, the bed height and the silicon conversion would have a similar effect on the process. Both of which embody the influence of the loading quantity. Under low silicon conversion, the reaction rate is liner relation with it^49^.

## Conclusion

CFD code MFIX with a validated chemistry model was used to describe the direct synthesis process. The direct synthesis of methylchlorosilane in the fluidized bed reactor was modeled. The effects of the general bed characteristics on the direct synthesis process were investigated. Several useful observation and conclusions were derived.

The effective reaction rate was found to depend on reaction temperature, particle diameter, superficial gas velocity and bed height, and the order is reaction temperature > particle diameter > bed height > superficial gas velocity. The M2 selectivity sharply decreases with the increase of the reaction temperature. The optimum temperature will take into account the reaction rate and M2 selectivity. The M2 selectivity is also badly affected by particle diameter, but less affected by other parameters.

The synthesis process is limited by mass transport in this system when the MeCl gas velocity is less than 8 times the minimum fluidization velocity; otherwise, it is limited by the surface reaction kinetics. The maximal influence of the above parameters on MeCl conversion is limited to less than 23%.

In general, the continuum model shows good agreement with the reaction rate and M2 selectivity. However, more physically based closures for e.g. the calculation of the frictional solids viscosity should be incorporated in the continuum models in the future. Benyahia et al.^33^ pointed out that using the kinetic theory of granular flow (KTGF) in which the k-epsilon model is not concluded is sufficient to model moderately dense flows. The continuum model should be simplified without the turbulence model. Improvements will follow to further investigate the applicability and flexibility of the model in the design and scale-up of fluidized bed reactors for synthesis process. The further development will also examine the influence of the reactor geometry, catalyst, and particle diameter distribution on hydrodynamics and direct synthesis reaction.

## Figures and Tables

**Table 1:** Comparisons between the reaction rates and M2 selectivity

Reactor type	Temperature (°C)	Particle diameter (dp, μm)	Catalyst	Reaction rate (gSilane/kgSi·h)	M2 selectivity (%)	Ref.
FBR	300	---	CuCl/Sn	100.5	---	7
FBR	320	---	10wt%Cu	80–100	90	10
FXBR	300	150–200	10wt%Cu	21.8	74.5	9
FBR	300	---	4.75wt%Cu	128.4	---	48
FXBR	310	---	10.2wt%Cu	280	---	32
FBR	300	150	10wt%Cu	126.4	81.0	Modeled(6U_mf_)
FBR	~300	Average ~ 150	10wt%Cu	135.8	79.2	Full-scale plant data measured
